# An Introduction to Systems Analytics and Integration of Big Omics Data

**DOI:** 10.3390/genes11030245

**Published:** 2020-02-26

**Authors:** Gary Hardiman

**Affiliations:** 1 School of Biological Sciences, Institute for Global Food Security (IGFS), Queen’s University Belfast, BT7 1NN Belfast Northern Ireland, UK; g.hardiman@qub.ac.uk; 2 Department of Medicine, Medical University of South Carolina, Charleston, SC 29425, USA

A major technological shift in the research community in the past decade has been the adoption of high throughput (HT) technologies to interrogate the genome, epigenome, transcriptome, and proteome in a massively parallel fashion [1,2]. This has provided both unique discovery opportunities and challenges for computational and quantitative scientists in predicting phenotypic outcomes. ‘Big Data’ encompasses the collection of data sets derived from technologies and so large and complex that their processing is impractical using traditional data processing applications. Challenges arise in collection, analysis, mining, sharing, transfer, visualization, archival and integration of Big Data. 

Genotype is one of three key factors that determine the phenotype, including inherited factors (DNA code), epigenetic factors (DNA methylation, histone modifications RNA-associated silencing) and non-inherited environmental factors [3]. In this special issue, there is a focus on systems level analysis of omics data, recent developments in pathway and network biology algorithm development, and integration of omics data with clinical and biomedical data using machine learning. The role of chromatin in genotype-phenotype is explored. Improvements to the Gene Ontology Resource to Facilitate More Informative Analysis and Interpretation of Alzheimer’s Disease Data is covered.

One of the pressing challenges for integrative computational biology and statistical genetics is predicting genotype-to-phenotype maps of organisms in the context of environmental influences. As noted in the collection perspective by Lewis Frey, genotypes and phenotypes realized in Omics data collections are linked through the various nuclear and cellular processes that convert encoded genotype information into a macroscale manifestation of the organism phenotype [4]. The ability to identify the key drivers of genotype to phenotype is challenging among the multitude of interacting molecules. Frey makes a compelling argument for the application of artificial intelligence (AI) that can automate computable phenotypes and integrate them with genotypes. Challenges need to be overcome namely the rapid growth of data, the inaccessibility of data through issues with incompleteness, inaccuracies, and heterogeneity and data silos. 

A review article in this collection by Núria Malats and colleagues explores the challenges that exist with the integration of Omics and Non-Omics (OnO) Data [5]. At present few omics-based algorithms that possess enough predictive ability are implemented in the clinic. Clinical/epidemiological data describe most of the variation in health-related traits. Effective modeling of this with omics data is urgently needed to increase the predictive ability of algorithms. Obstacles in OnO data integration are the nature and heterogeneity of non-omics data, the relationship between OnO data termed ascertainment bias, the presence of interactions, the fairness of the computational models, and the presence of sub-phenotypes. Most data to date is focused on RNA expression data and studies have incorporated non-omics data in a low-dimensionality manner. Integrative strategies typically adopt one of three modeling methods: Independent, conditional, or joint modeling. Joint modeling, where omics and non-omics data are modelled together in a supervised or unsupervised manner, are preferred for integrating large-scale OnO data, as they account for the correlation structure between the two data types. Additionally, they provide greater complexity than conditional or independent modeling [5].

Data from different sources (e.g., genome, epigenome, transcriptome, proteome, metabolome) tends to be analyzed in isolation using statistical and machine learning (ML) methods. Effective data integration poses new computational challenges [6]. State-of-the-art ML-based approaches for tackling five specific computational challenges associated with integrative analysis: namely the curse of dimensionality, data heterogeneity, missing data, class imbalance and scalability issues are reviewed by Peipei Ping and colleagues. Anagha Joshi and colleagues review Genotype to Phenotype via Chromatin [7]. They note that mapping mutations to causal genes and therapeutic targets to date has been quite limited. The majority of disease-associated mutations lie in inter-genic regions. An emerging trend is thus to focus on the epigenetic control of the disease to generate more complete functional genomic maps. Recent studies unravelling the mechanistic understanding of epigenetic processes in disease development and progression are reviewed [7].

This special issue presented new methodologies in the context of gene-environment, tissue-specific gene expression and how external factors or host genetics impact the microbiome [8,9,10]. Wolf and colleagues developed an analytical approach for identifying the main effects and interactions between genetic and environmental factors linked to a disease outcome [8]. The method involves selection of candidate genetic and/or environmental factors, utilization of a machine learning algorithm Logic Forest to identify the salient effects and interactions in the disease, followed by confirmation of the association between interactions identified by the algorithm using logistic regression. A case study examining the association between SNPs and cigarette smoke exposure with risk of developing systemic lupus erythematosus (SLE) is presented. This identified genetic and environmental risk factors, and potential interactions between exposure to secondhand smoke as a child and genetic variation in the Integrin alpha M (*ITGAM*) gene associated with increased risk of SLE [8].

Cai and colleagues exploited transcriptomic data from multiple tissues generated by the Genotype-Tissue Expression (GTEx) project [10,11] and developed a new methodology that integrates machine learning algorithms to identify genes widely expressed in human body tissues with different expression signatures that can distinguish different tissue types. The approach allows tissue classification via a 432 gene signature of quantitatively tissue-specific expression, suggesting that these genes could also play important roles in tissue development and function [10].

Three notable dynamic interactions play a role in phenotypic outcome. The first, is the association between the environment and the host; the second is that between the microbiome and host health or disease state; and the third is the linkage between the environment and the microbiome. Owing to this complexity the majority of observational and experimental study designs fail to fully assess the direct causal roles of the microbiome. To address this Big Omics challenge, Alekseyenko and colleagues developed a framework for multivariate omnibus distance mediation analysis (MODIMA). They exploited the power of energy statistics, to facilitate analysis of multivariate exposure-mediator-response triples [9]. 

An important resource for Big Omics data analysis is the Gene Ontology (GO, geneontology.org) which is used when performing gene enrichment analysis. Ruth Lovering and colleagues at University College London (UCL) describe improvements to the GO Resource to improve analysis and interpretation of Alzheimer’s Disease data [12]. This project, funded by the Alzheimer’s Research United Kingdom foundation and led by the UCL biocuration team, enhanced the GO resource by developing new neurological GO terms, and annotating gene products associated with dementia. Of the total 2055 annotations contributed for the prioritized gene products, 526 had associated proteins and complexes with neurological GO terms. To ensure that these descriptive annotations could be provided for Alzheimer’s-relevant gene products, over 70 new GO terms were created. This important novel resource will benefit the scientific community and enhance the interpretation of dementia data [12].

Functional enrichment analyses often result in long lists of biological terms associated to proteins that can be difficult to digest and interpret. Fiero and colleagues addressed this Big Omics data analysis challenge via the development of Network-based Visualization for Omics (NeVOmics).This tool provides a hypergeometric distribution test to compute significantly enriched biological terms. It enables analysis of cluster distribution and relationship of proteins to biological processes and pathways [13]. Even though databases such as the Cancer Cell Line Encyclopedia (CCLE), the Cancer Therapeutics Response Portal (CTRP), and The Cancer Genome Atlas (TCGA) are available it remains challenging for researchers to explore the relationship between drug response and the underlying genomic features due data heterogeneity. Sung Min Ahn and colleagues address this via the development of the Integrated Pharmacogenomic Database of Cancer Cell Lines and Tissues (IPCT) [14]. The IPCT allows users to identify new linkages between drug responses and genomic features. It also allows comparison of the genomic features of sensitive cell lines or small molecules with the genomic features of tumor tissues.

30% of all genes in mammalian cells are predicted to be regulated by microRNA (miRNAs) miRNAs. Da Silveira and Renaud and colleagues describe a new tool, “miRmapper”, which identifies the most dominant miRNAs in a miRNA–mRNA network and recognizes similarities between miRNAs based on commonly regulated mRNAs. The most relevant miRNAs are not necessarily those with the greatest change in expression levels between healthy and diseased tissue. Differentially expressed (DE) miRNAs that modulate a large number of messenger RNA (mRNA) transcripts ultimately have a greater influence in determining phenotypic outcomes and are more important in a global biological context than miRNAs that modulate just a few mRNA transcripts. Da Silveira and Renaud exploit this concept to analyze data from a nonmetastatic and metastatic bladder cancer cell lines and demonstrated that the most relevant miRNAs in a cellular context are not necessarily those with the greatest fold change [15]. 

In summary, the emergence and global utilization of high throughput (HT) technologies, including deep sequencing technologies (genomics) and mass spectrometry (proteomics, metabolomics, lipids), has allowed geneticists, biologists, and biostatisticians to bridge the gap between genotype and phenotype on a scale that was not possible previously. In this special issue integration strategies for systems level analysis of Omics data, recent developments in gene ontology pathway and network algorithm development are explored as is the integration of Omics data with clinical and biomedical data.
